# Early family regularity protects against later disruptive behavior

**DOI:** 10.1007/s00787-015-0797-y

**Published:** 2015-11-20

**Authors:** Jolien Rijlaarsdam, Henning Tiemeier, Ank P. Ringoot, Masha Y. Ivanova, Vincent W. V. Jaddoe, Frank C. Verhulst, Sabine J. Roza

**Affiliations:** The Generation R Study Group, Erasmus MC-University Medical Center Rotterdam, Rotterdam, The Netherlands; Department of Child and Adolescent Psychiatry/Psychology, Erasmus MC-University Medical Center Rotterdam, Rotterdam, The Netherlands; Centre for Child and Family Studies, Leiden University, Leiden, The Netherlands; Department of Epidemiology, Erasmus MC-University Medical Center Rotterdam, Rotterdam, The Netherlands; Department of Psychiatry (Dp-1404), Erasmus MC-University Medical Center Rotterdam, Postal Box 2040, 3000 CA Rotterdam, The Netherlands; Department of Psychiatry, University of Vermont, Burlington, VT USA; Department of Paediatrics, Erasmus MC-University Medical Center Rotterdam, Rotterdam, The Netherlands

**Keywords:** Family regularity, Infant temperament, Child disruptive behavior, Prospective study

## Abstract

Infants’ temperamental anger or frustration reactions are highly stable, but are also influenced by maturation and experience. It is yet unclear why some infants high in anger or frustration reactions develop disruptive behavior problems whereas others do not. We examined family regularity, conceptualized as the consistency of mealtime and bedtime routines, as a protective factor against the development of oppositional and aggressive behavior. This study used prospectively collected data from 3136 families participating in the Generation R Study. Infant anger or frustration reactions and family regularity were reported by mothers when children were ages 6 months and 2–4 years, respectively. Multiple informants (parents, teachers, and children) and methods (questionnaire and interview) were used in the assessment of children’s oppositional and aggressive behavior at age 6. Higher levels of family regularity were associated with lower levels of child aggression independent of temperamental anger or frustration reactions (*β* = −0.05, *p* = 0.003). The association between child oppositional behavior and temperamental anger or frustration reactions was moderated by family regularity and child gender (*β* = 0.11, *p* = 0.046): family regularity reduced the risk for oppositional behavior among those boys who showed anger or frustration reactions in infancy. In conclusion, family regularity reduced the risk for child aggression and showed a gender-specific protective effect against child oppositional behavior associated with anger or frustration reactions. Families that ensured regularity of mealtime and bedtime routines buffered their infant sons high in anger or frustration reactions from developing oppositional behavior.

**Electronic supplementary material:**

The online version of this article (doi:10.1007/s00787-015-0797-y) contains supplementary material, which is available to authorized users.

## Introduction

Children’s temperamental anger or frustration reactions, defined as negative affect in reaction to interruption of ongoing tasks or blocking the child’s goal, are already observable in the first 2–3 months of life, and may decline across early childhood as a function of maturation and experience [1, 2]. However, some children retain their anger or frustration reactions across early childhood and develop disruptive behavior problems, such as oppositional behavior and aggression [3, 4]. These disruptive behavior problems, in turn, are considered primary precursors to conduct problems and the development of antisocial personality disorder [5, 6]. The identification of protective factors that buffer the association between infant temperamental anger or frustration reactions and later disruptive behavior problems is important for the development of intervention strategies.

It has been repeatedly demonstrated that family regularity plays an important role in the behavioral development of children [7]. The definition of family regularity has evolved in the past two decades to include the consistency of day-to-day family routines that occur at mealtimes or bedtimes [8–11] rather than just global or distal aspects of the family environment, such as changes in parental marital status or household composition. A family’s adherence to meal- and bed-time routines, as retrospectively reported by college students about their family of origin, has been found to buffer individuals with a pessimistic attributional style from developing depressive symptoms [9]. Greater attention to a family’s adherence to routines may be profitable, as it represents an accessible and modifiable target for interventions to reduce or prevent disruptive behavior problems in children.

The protective function of family regularity is thought to be due to an increased sense of predictability, controllability and consistency in children’s lives. Specifically, caregivers who ensure a family environment with predictable day-to-day routines and rules may foster their children’s self-control skills, including their ability to control their emotions and behavior [7, 8, 11]. Conversely, caregivers who do not adhere to consistent, predictable family routines may exacerbate their children’s fear, anger or frustration reactions, leading to higher levels of emotional or behavior problems. An important research question that has yet to receive empirical attention is whether consistent family routines protect infants high in anger or frustration reactions from developing disruptive behavior problems.

Using data from a large population-based prospective cohort, we explored the association of infants’ anger or frustration reactions with later disruptive problems at age 6, and moderation of this association by family regularity. Several subtypes of disruptive behaviors were investigated, including aggression and oppositional-defiance, because of their distinct developmental trajectories [12]. We hypothesized that infants high in temperamental anger or frustration reactions tend to develop disruptive behavior in early childhood and may benefit from more predictable routines and rules in fostering their ability to master their sensations of irritability or anger, weakening the association between infant temperamental dispositions and later oppositional behavior and aggression. Furthermore, in light of differential developmental trajectories for disruptive behavior in males versus females [13], potential gender differences were examined. There is evidence suggesting that more males compared to females follow an early onset persistent trajectory of disruptive behavior (i.e., manifest disruptive behaviors starting early and persisting over time) [13]. The vast majority of the females are thought to have an adolescence-onset of disruptive behavior. Protective effects in childhood, including those of family regularity, may be strongest among boys, who are at the highest risk for disruptive behavior [14, 15].

## Method

### Participants

The present research was conducted within the framework of the Generation R Study, a population-based cohort from fetal life onwards. Sample ascertainment and participation have been described in detail elsewhere [16]. Pregnant women living in the study area in Rotterdam, the Netherlands, with an expected delivery date between April 2002 and January 2006 were invited to participate. The study was conducted in accordance with the guidelines proposed by the World Medical Association Declaration of Helsinki and was approved by the Medical Ethical Committee of the Erasmus University Medical Center, Rotterdam. Written consent was obtained from all participants.

In the Generation R Study, 4119 children had data on temperamental anger or frustration at age 6 months. Of these children, 3741 had mother reports on family bedtime routines at age 2 years and mealtime routines at age 4 years. All 3136 children who also had at least two out of three informant (parent, teacher, or child) ratings on oppositional behavior or aggression at age 6 years were included in the current analysis. Table 1 shows descriptive statistics for the sample. Mothers of children not included (*n* = 983) were more often lower educated (26.2 vs. 12.8 %, *χ*^2^(1) = 118.61, *p* < 0.001) and were more often single (12.5 vs. 8.3 %, *χ*^2^(1) = 14.66, *p* < 0.001) than mothers of children included (*n* = 3136).

**Table 1:** Sample characteristics (*n* = 3136)

Infant anger or frustration reactions, score	0.68 (0.32)*
Family regularity, score	0.08 (−1.55, 0.34)
Child oppositional behavior, parent report score	2.00 (0.00–12.00)*
Child oppositional behavior, teacher report score	0.00 (0.00–10.00)*
Child oppositional behavior, child report score	14.00 (7.00–31.00)*
Child aggression, parent report score	4.00 (0.00–33.78)*
Child aggression, teacher report score	0.00 (0.00–34.00)*
Child aggression, child report score	49.00 (32.00–120.00)*
Maternal psychopathology symptoms, high (%)	6.3
Maternal age in years	31.66 (4.46)
Maternal education, low (%)	12.8
Maternal smoking during pregnancy, yes (%)	11.2
Family income, low (%)	9.1
Marital status, single (%)	8.3
Parity, multiparous (%)	40.3
Child national origin, non-Western (%)	23.2
Child gender, boy (%)	49.4

Values represent mean (standard deviation) for continuous normally distributed variables and median (range) for continuous non-normally distributed variables

* Boys show higher rates than girls (*p* < 0.05). *P* values are derived from independent sample *t* tests for continuous normally distributed variables, Mann–Whitney–Wilcoxon tests for continuous non-normally distributed variables, and Chi square tests for categorical variables

### Measures

#### Infant anger or frustration reactions

Infants’ anger or frustration reactions were assessed at age 6 months using an adapted version of the Infant Behaviour Questionnaire—Revised (IBQ-R) [17] which has been described in detail previously [18]. The IBQ Distress to Limitations subscale refers to negative emotionality and reaction to frustrating situations. Mothers rated the extent to which each statement described their infant in the previous weeks on a 3-point scale (0 = never, 1 = sometimes, 2 = often) [17]. In a pilot study, 3 of the 16 items belonging to the distress to limitation subscale were eliminated due to content considerations (i.e., mothers judged them as overlapping with other items). The remaining 13 items of the scale showed acceptable internal consistency in this sample (Cronbach’s alpha = 0.74).

#### Family regularity

Multiple domains of family regularity were assessed, including bedtime routines (i.e., whether or not the study child has gone to bed in the evening at around the same time and whether or not parents have a set pattern or ritual with their children at bedtime) at age 2 years, and family meal location (i.e., frequency per week of having breakfast or dinner around the table together with the family) and the child’s meal frequency (i.e., frequency per week having breakfast, lunch, and evening meals) at age 4 years. CFA was employed in Mplus version 7.11 [19] to combine these items into a single construct to represent family regularity. The default weighted least squares means and variance adjusted (WLSMV) estimator for categorical data was used, which handles missing data using the pairwise present procedure (i.e., uses all available information). Model fit was established using the root mean square error of approximation (RMSEA; acceptable fit ≤0.08) as well as the comparative fit index and the Tucker–Lewis index (CFI and TLI; acceptable fit ≥0.90) [20]. The domains of family regularity and accompanying items, together with their response scales, are shown in supplementary Table S1.

#### Child disruptive behavior problems

Parents completed the child behavior checklist (CBCL/1.5–5) (mean age = 6.03, SD = 0.39), from which the 6-item oppositional defiant scale and the 19-item aggression scale were used [21]. Teachers completed the teacher report form (TRF) (mean age = 6.75 years, SD = 1.30), from which the 5-item oppositional defiant scale and the 20-item aggression scale were used [22]. Informants rated the extent to which each statement described the child “now or within the past 2 months” on a 3-point scale ranging from 0 (not true*)* to 2 (very true or often true). Good reliability and validity have been reported for both the CBCL and the TRF [21, 22].

We also used the Berkeley puppet interview (BPI) [23, 24], a validated semi-structured interactive interview technique, to obtain self-reports from young children (mean age = 6.11 years, SD = 0.41). In line with the procedures outlined by the developers, the interviews were taped for scoring by coders. The BPI has shown an adequate factor structure, acceptable internal consistencies and validity as indexed by associations with socio-demographic factors [25]. From the BPI, we used the 5-item oppositional defiant scale and the 21-item externalizing scale that showed overlap with the CBCL and TRF aggression scales. All BPI items were rated on a 7-point scale ranging from 1 (very positive) to 7 (very negative).

Parent, teacher and self-reports of children’s oppositional behavior or aggression were aggregated to obtain multiple-informant scores. The single informant scores were first residualized for age to account for the variable assessment points and z-standardized. Following recommendations in the literature [26], we conducted an unrotated principal component analysis on the scales using three fixed components. The first component weights the parent, teacher and child reported scores in the same direction and provides a multiple informant measure that is relatively free of information bias. Conversely, the second and third components are thought to reflect differences in context (home versus school) and perspective (other versus self) between the respondents. We went forward with a simpler approach of averaging the three residualized and z-standardized single informant scores, because the correlation with the multiple informant component coefficient was perfect (*r* = 1.00 for each of the two behavioral outcomes). Pearson correlations between the multiple and single informant scores ranged from *r* = 0.65 to *r* = 0.72 for oppositional behavior, and from *r* = 0.68 to *r* = 0.74 for aggression. The correlation between the multiple informant scores of oppositional behavior and aggression was *r* = 0.87.

#### Family background characteristics

Information on child gender (0 = boy; 1 = girl) was obtained from the midwife and hospital registries at birth. We included as covariates several child and parental characteristics assessed by questionnaire during pregnancy: family income (<€1200 versus ≥€1200), marital status (single versus married or cohabiting), maternal age at intake, maternal tobacco smoking during pregnancy (did not smoke as soon as pregnancy was known versus continued smoking during pregnancy), parity (previous pregnancies: 0 versus ≥1), maternal education (primary school or lower vocational training versus higher), and child national origin (Western versus non-Western) [27]. At 20 weeks of pregnancy, mothers were asked to complete the well-validated brief symptom inventory (BSI) [28]. Maternal psychopathology was defined on the basis of the BSI global symptom inventory as either “high” or as “not high” [29].

### Statistical analyses

We used multiple linear regression analyses with product terms in SPSS version 21 (IBM Corporation) to examine the associations of infant anger or frustration reactions with childhood disruptive behavior, and moderation of these associations by family regularity and child gender. For each of the two dependent variables (oppositional behavior and aggression), several regression models were run. In the first step, we entered infant anger or frustration reactions, family regularity, child gender, and covariates. In the second step, we entered two-way interactions (anger or frustration reactions × family regularity, anger or frustration reactions × gender, family regularity × gender). In the third step, we entered the three-way interaction (anger or frustration reactions × family regularity × gender). Children’s disruptive behavior scores were logarithmic (Log 10) transformed prior to regression analysis to approximate a normal distribution. In the same vein, reflect and inverse transformations were applied to the family regularity scores. All independent variables were mean centered to control for multicollinearity. In a sensitivity analysis, we combined (i.e., averaged) the aggression and oppositional behavior scores into a more general behavioral score and rerun the regression models.

Missing values on covariates (percentages of missing ranged from 0.1 to 17.4 %, with maternal psychopathology having the highest missing rate) were handled by use of the Markov Chain Monte Carlo multiple imputation technique with predictive mean matching for continuous variables in SPSS. A total of 10 datasets were generated and parameter estimates were averaged over the set of analyses.

## Results

**Fig. 1:**
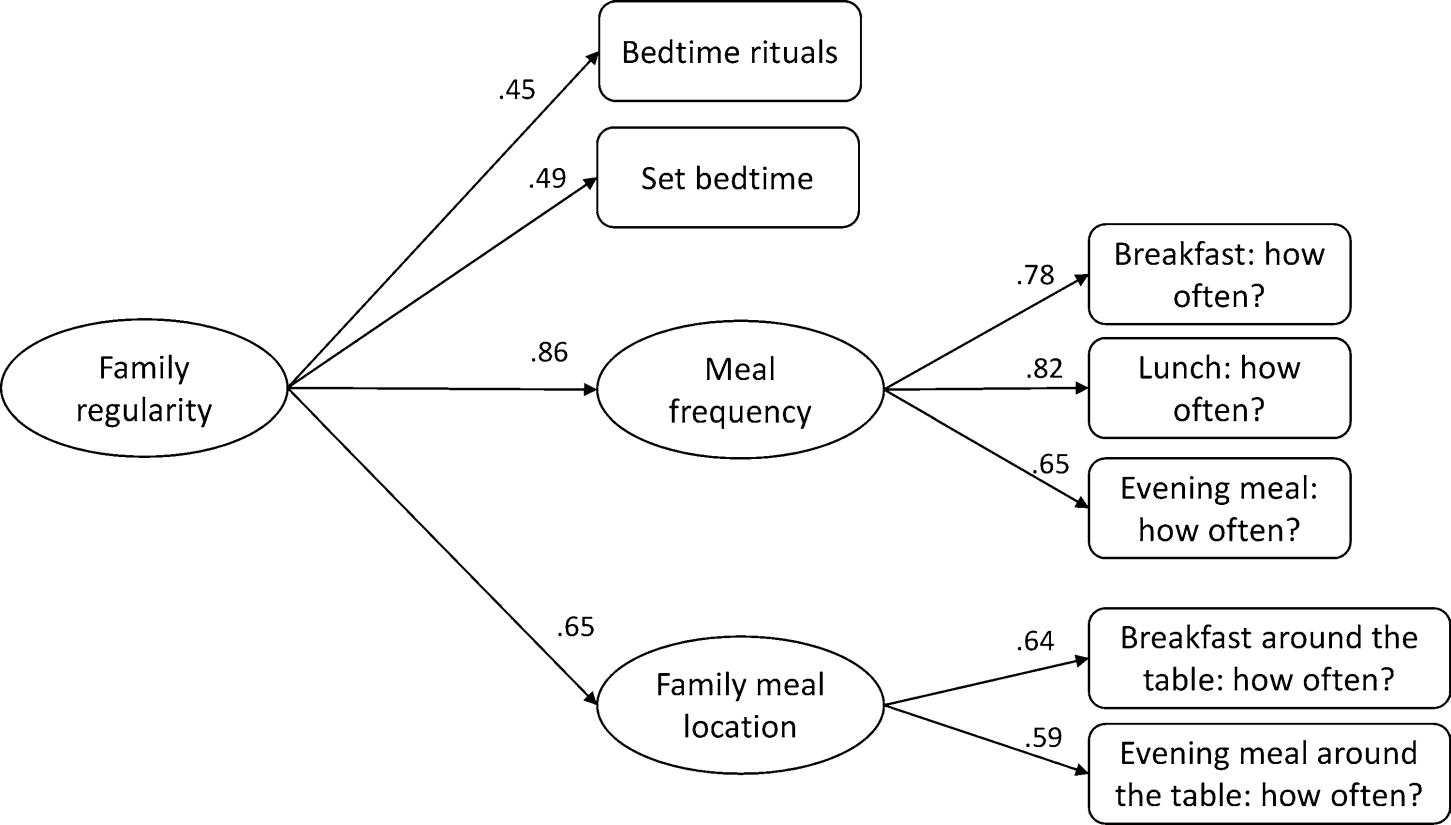
Confirmatory factor analysis model of family regularity. Values represent standardized factor loadings for the second-order confirmatory factor analysis of family regularity. All were statistically significant (*p* < 0.001)

Results from CFA supported the second-order measurement model of family regularity (RMSEA = 0.05; CFI = 0.94; TLI = 0.89; *χ*^2^(12) = 169.44). Factor loadings were all relatively high and statistically significant (see Fig. 1), and the resulting factor score was used in all subsequent analyses to represent family regularity. Table 2 displays the correlations among study variables. Family regularity correlated with infant anger or frustration reactions (*r* = −0.10, *p* < 0.05), child oppositional behavior (*r* = −0.04, *p* < 0.05), child aggression (*r* = −0.09, *p* < 0.05) and all family background variables (range *r* = –0.23 to 0.11, all *p* < 0.05) but gender.

**Table 2:** Correlations among study variables

	1	2	3	4	5	6	7	8	9	10	11	12	13
1. Infant anger or frustration, score	–	**−0.10**	**0.09**	**0.12**	**0.17**	**−0.07**	**0.07**	0.01	**0.16**	**0.10**	**0.07**	**0.25**	**−0.07**
2. Family regularity, score		–	**−0.04**	**−0.09**	**−0.12**	**0.11**	**−0.17**	**−0.11**	**−0.18**	**−0.15**	**0.04**	**−0.23**	−0.01
3. Child oppositional, score			–	**0.87**	**0.07**	0.00	0.01	**0.05**	0.02	**0.08**	**−0.04**	−0.01	**−0.16**
4. Child aggression, score				–	**0.11**	**−0.04**	**0.07**	**0.06**	**0.09**	**0.12**	**−0.05**	**0.07**	**−0.22**
5. Maternal psychopathology, high					–	**−0.16**	**0.16**	**0.15**	**0.31**	**0.14**	0.01	**0.22**	−0.03
6. Maternal age, years						–	**−0.21**	**−0.06**	**−0.26**	**−0.12**	**0.27**	**−0.19**	−0.01
7. Maternal education, low							–	**0.21**	**0.37**	**0.17**	**0.06**	**0.27**	0.01
8. Maternal smoking, yes								–	**0.11**	**0.13**	0.02	**0.07**	0.00
9. Family income, low									–	**0.43**	**0.05**	**0.40**	0.01
10. Marital status, single										–	**−0.06**	**0.25**	−0.02
11. Parity, multiparous											–	**0.07**	0.01
12. Child national origin, non-western												–	0.01
13. Child gender, girl													–

Pearson’s *r* coefficients are reported for correlations with continuous variables, and point-biserial coefficients for correlations with continuous and dichotomous variables. Variables highlighted in bold were statistically significant at *p* < 0.05

Table 3 shows that infant anger or frustration reactions were associated with both child oppositional behavior and aggression (*β* = 0.08, *p* < 0.001). Family regularity was associated with child aggression (*β* = −0.05, *p* = 0.003) but not oppositional behavior (*β* = −0.03, *p* = 0.093), independent of infant anger or frustration reactions and gender. Additionally, the three-way interaction of anger or frustration reactions × family regularity × gender was significant (*β* = 0.11, *p* = 0.046) in the analysis of child oppositional behavior and marginally significant in the analysis of child aggression (*β* = 0.10, *p* = 0.051). In the sensitivity analysis on the more general behavioral score, the regression coefficients of the family regularity main effect (*β* = −0.04, *p* = 0.016) and the three-way interaction (*β* = 0.11, *p* = 0.041) were statistically significant and similar to those obtained for the individual aggression and oppositional behavior scores.

**Table 3:** Associations between infant anger or frustration reactions, family regularity, and disruptive behavior at age 6

	Children’s oppositional behavior, score (*n* = 3136)			
	*B*	95 % CI	*β*	*p* value
Step 1: Infant anger or frustration reactions, score	0.03	0.017; 0.045	0.08	<0.001
Child gender^a^	−0.04	−0.045; −0.028	−0.15	<0.001
Family regularity, score	−0.02	−0.045; 0.003	−0.03	0.093
Step 2: Infant anger or frustration reactions, score	0.04	−0.007; 0.077	0.09	0.103
Child gender^a^	−0.04	−0.045; −0.028	−0.15	<0.001
Family regularity, score	−0.04	−0.113; 0.036	−0.06	0.315
Infant anger or frustration reactions × family regularity	−0.03	−0.099; 0.045	−0.01	0.461
Infant anger or frustration reactions × gender	−0.003	−0.029; 0.024	−0.01	0.848
Family regularity × gender	0.01	−0.035; 0.059	0.02	0.622
Step 3: Infant anger or frustration reactions, score	0.04	−0.008; 0.077	0.09	0.108
Child gender^a^	−0.04	−0.044; −0.027	−0.15	<0.001
Family regularity, score	−0.03	−0.107; 0.043	−0.05	0.397
Infant anger or frustration reactions × family regularity	−0.24	−0.467; −0.019	−0.12	0.033
Infant anger or frustration reactions × gender	−0.002	−0.029; 0.025	−0.01	0.874
Family regularity × gender	0.01	−0.038; 0.056	0.02	0.713
Infant anger or frustration reactions × family regularity × gender	0.15	0.003; 0.289	0.11	0.046

	Children’s aggression, score (*n* = 3135)			
	*B*	95 % CI	*β*	*p* value
Step 1: Infant anger or frustration reactions, score	0.03	0.016; 0.042	0.08	<0.001
Child gender^a^	−0.05	−0.058; −0.042	−0.21	<0.001
Family regularity, score	−0.03	−0.057; −0.012	−0.05	0.003
Step 2: Infant anger or frustration reactions, score	0.05	0.006; 0.085	0.12	0.024
Child gender^a^	−0.05	−0.058; −0.042	−0.21	<0.001
Family regularity, score	−0.07	−0.142; −0.002	−0.11	0.045
Infant anger or frustration reactions × family regularity	−0.02	−0.085; 0.051	−0.01	0.622
Infant anger or frustration reactions × gender	−0.01	−0.036; 0.014	−0.05	0.396
Family regularity × gender	0.03	−0.019; 0.069	0.06	0.268
Step 3: Infant anger or frustration reactions, score	0.05	0.005; 0.085	0.12	0.026
Child gender^a^	−0.05	−0.057; −0.041	−0.21	<0.001
Family regularity, score	−0.07	−0.136; 0.004	−0.10	0.065
Infant anger or frustration reactions × family regularity	−0.21	−0.424;−0.005	−0.11	0.045
Infant anger or frustration reactions × gender	−0.01	−0.035; 0.015	−0.04	0.414
Family regularity × gender	0.02	−0.022; 0.066	0.05	0.325
Infant anger or frustration reactions × family regularity × gender	0.13	−0.001; 0.267	0.10	0.051

*B* (unstandardized regression coefficients) and *β* (standardized regression coefficients) are derived from the indicated steps of the regression model. All models are adjusted for child national origin, parity, maternal psychopathology, maternal smoking during pregnancy, marital status, family income, maternal education, and maternal age. Multiple informants (parent, teacher, child) were used in the assessment of child disruptive problems. The single informant scores were residualized for age to account for the variable assessment points, z-standardized, and averaged

^a^Reference category is boys

**Fig. 2:**
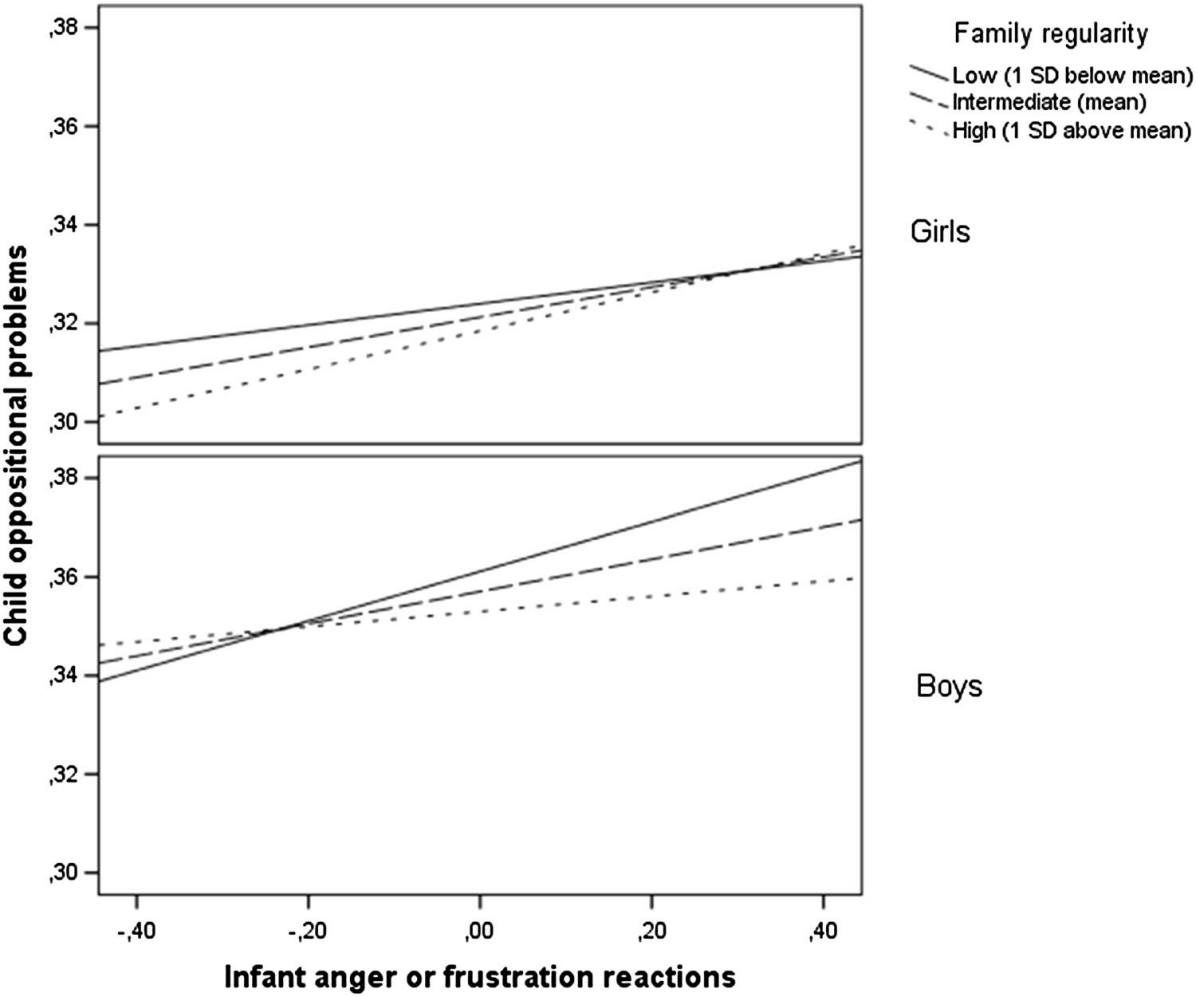
The association between infant anger or frustration reactions and childhood oppositional behavior in boys and girls as moderated by family regularity

To probe the significant three-way interaction, we estimated simple slopes for the association between infant anger or frustration reactions and oppositional problems using the SPSS PROCESS macro developed by Hayes [30]. First, simple slopes were estimated for the interaction of this association by family regularity at values of child gender (boys versus girls). Among boys, the infant anger or frustration reactions × family regularity interaction was marginally significant (*b* = −0.10, *p* = 0.06). Among girls, this interaction was in the opposite direction, but not significant (*b* = 0.05, *p* = 0.36). Second, simple slopes were estimated for combinations of low (1 SD below mean) versus high (1 SD above mean) values of family regularity and child gender. Among boys, infant distress to limitations was associated with oppositional behavior at low (*b* = 0.05, *p* < 0.001) but not at high values of family regularity (*b* = 0.01, *p* = 0.27), suggesting a classic protective effect. Among girls, infant distress to limitations was associated with oppositional behavior at high (*b* = 0.04, *p* = 0.01) but not at low (*b* = 0.02, *p* = 0.12) values of family regularity. However, as indicated above, the differences in associations were significant only in boys (when compared with girls). These associations are depicted in Fig. 2.

## Discussion

This large prospective population-based cohort study examined family regularity, conceptualized as the consistency of day-to-day family routines, as a protective factor against children’s disruptive behavior. Family regularity at age 2–4 years reduced the risk for child aggression at age 6 years independently of temperamental anger or frustration reactions at age 6 months. Furthermore, family regularity reduced the risk for oppositional behavior at age 6 years among those boys who showed anger or frustration reactions in infancy.

The current findings corroborate previous research indicating that family regularity is important and serves a protective function among children at risk for developing psychopathology. The importance of family regularity for successful child behavioral development had been previously reported in the context of parental depressive symptoms in a cross-sectional clinical sample [8]. The current study extends these findings to a large prospective population-based sample and also evaluates the gender-specific protective role of family regularity using interaction terms. Because interactions are difficult to detect in non-experimental studies, large sample sizes are needed and even small effect sizes may have clinical significance [31]. When we consider the three-way interaction of anger or frustration reactions × family regularity × gender and the main effect of family regularity, the standardized regression coefficients were small and very similar in magnitude for the two behavioral outcomes. The three-way interaction was statistically significant only for oppositional behavior, while the family regularity main effect was statistically significant only for aggression. However, standardized regression coefficients for the aggregate disruptive behavior score were statistically significant and similar in magnitude to those observed for the individual scores, suggesting that the findings reflect shared variance rather than variance due to solely aggression or oppositional behavior.

Infant anger or frustration reactions at age 6 months were prospectively related to both oppositional behavior and aggression at age 6 years for both boys and girls, as indicated by significant main effects and non-significant interactive effects with gender. Interestingly, a protective effect of family regularity against child oppositional problems associated with infant anger or frustration reactions was observed only among boys (through a significant anger or frustration reactions × family regularity × gender interaction). The protective function of family regularity might be explained by processes of modeling and social learning [32]. For example, infants may observe their parents set the table and gathering family members to attain regularity in mealtime routines, learning that rules and boundaries can be established and that behavior can be organized accordingly [11].

Although speculative, the finding of a gender-specific protective function of family regularity may be due to differential developmental trajectories of disruptive behavior (e.g., aggression, opposition, delinquent and criminal behaviors). Whereas more males compared to females follow an early-onset persistent trajectory (i.e., manifest antisocial behaviors starting early and persisting over time), an adolescence-delayed-onset trajectory is considered more appropriate to characterize females’ disruptive behavior [13]. Females following an adolescence-delayed-onset trajectory are hypothesized to have childhood risk factors (e.g., family risk factors) and adult consequences (e.g., persistence of antisocial behaviors) similar to early onset males, but a delayed onset of disruptive behavior. This delayed onset could be explained by socialization processes that discourage girls from aggression and encourage the “channeling” of girls’ early problem behavior into predominantly emotional problems [33], leading to the inhibition of childhood disruptive behavior even for females exposed to risk factors. Interestingly, these developmental trajectories have been found to vary across different types of behavior (e.g., aggression, opposition). Further research is needed to investigate the extent to which the observed gender difference in a buffering effect of family regularity extends into adolescence.

A strength of the current study is the prospective population-based design, as well as the multiple informants (child, parent, and teacher) and methods (questionnaire and interview) that were used in the assessment of children’s disruptive behavior. Thus, the current findings may be more easily generalizable to the broader population, and are less likely an artifact of reporter bias. Of note, selection occurred toward well-functioning families with higher socio-economic status. Although selective drop-out might have reduced statistical power, it does not necessarily affect the validity of regression models predicting disruptive behavior problems [34]. The fact that children’s anger and frustration reactions (age 6 months), family regularity (age 2–4 years), and disruptive behavior problems (age 6 years) were all assessed at different time points adds to the robustness of our findings. Family regularity is a complex construct that challenges a simple definition and requires extensive study regarding its correlates. The low to moderate correlations observed in this study suggest that family regularity is at least partially distinct from family background characteristics, such as socio-economic status, national origin, marital status, and maternal psychopathology.

In conclusion, family regularity reduced the risk for child aggression and showed a gender-specific protective effect against child oppositional behavior associated with temperamental anger or frustration reactions. Families that ensured regularity of mealtime and bedtime routines protected their infant sons high in anger or frustration reactions from developing oppositional behavior. Although the adverse effects of temperamental anger or frustration reactions may be somewhat difficult to address with an intervention, they may be successfully buffered by helping parents to adhere to consistent, predictable day-to-day family routines. For example, the evidence-based Video-feedback Intervention to promote Positive Parenting (VIPP) may help reduce or prevent disruptive behavior problems in children by improving environmental stability and parental sensitivity [35]. Importantly, this study examined the regularity of family routines and not child neglect or abuse. The finding that the protective function of family regularity was independent of contextual risk and maternal psychopathology is promising in that family mealtime and bedtime routines may be more easily controlled by the parent or child [10]. The findings of this study may further our understanding of resiliency and provide a platform for intervention research to prevent the emergence of disruptive behavior problems.

## Electronic supplementary material

Below is the link to the electronic supplementary material.

Supplementary material 1 (DOCX 11 kb)
