# Supplementary material for: Early family regularity protects against later disruptive behavior
Source: Eur Child Adolesc Psychiatry. 2015 Nov 20;25:781–9. doi: 10.1007/s00787-015-0797-y (PMC4932141; doi:10.1007/s00787-015-0797-y)
Supplement: Supplementary file 1 — Supplementary material 1 (DOCX 11 kb) [file 787_2015_797_MOESM1_ESM.docx]

**Supplementary Table S1** Family regularity items

| Family meal frequency |  |
| --- | --- |
| How often does your child eat breakfast? |  |
| % Never | 0.5 |
| % 1-2 days per week | 1.3 |
| % 3-4 days per week | 2.2 |
| % 5-6 days per week | 3.7 |
| % Every day | 92.3 |
| How often does your child eat at lunch time? |  |
| % Never | 0.3 |
| % 1-2 days per week | 0.5 |
| % 3-4 days per week | 1.5 |
| % 5-6 days per week | 5.0 |
| % Every day | 92.7 |
| How often does your child have an evening meal? |  |
| % Never | 0.1 |
| % 1-2 days per week | 0.6 |
| % 3-4 days per week | 1.6 |
| % 5-6 days per week | 4.5 |
| % Every day | 93.2 |
| Family meal location |  |
| How often do you have breakfast around the table together with your child/children? |  |
| Never | 6.3 |
| % 1-2 days per week | 17.9 |
| % 3-4 days per week | 15.1 |
| % 5-6 days per week | 9.9 |
| % Every day | 50.8 |
| How often do you have the evening meal around the table together with your child/children? |  |
| Never | 0.8 |
| % 1-2 days per week | 2.0 |
| % 3-4 days per week | 5.7 |
| % 5-6 days per week | 13.0 |
| % Every day | 78.4 |
| Bedtime routines |  |
| Do you have a set pattern or ritual with your child at bedtime (% yes)? | 90.0 |
| Has your child gone to bed in the evening at around the same time (% yes)? | 83.1 |
